# Future of supplementary private hospitalization insurance in Switzerland: a policy brief

**DOI:** 10.3389/phrs.2026.1609046

**Published:** 2026-09-25

**Authors:** Kateryna Riabchenko, Carlo De Pietro

**Affiliations:** Department of Business Economics, Health and Social Care, University of Applied Sciences and Arts of Southern Switzerland, Manno, Switzerland

**Keywords:** hospital financing, inpatient costs, private insurance, Switzerland, voluntary hospitalization insurance

## Abstract

**Background:**

Voluntary hospitalization insurance (VHI) in Switzerland has lost part of its traditional role as mandatory health insurance has expanded, hospital financing reforms have increased public co-financing of inpatient care, and patterns of hospitalization have changed. Nevertheless, VHI remains strategically important for hospitals, insurers, and some providers.

**Analysis:**

This policy brief draws on a structured review of academic and grey literature, regulatory and policy documents, market information, and ten semi-structured interviews with key stakeholders. The analysis identifies four main challenges: growing dependence on mandatory health insurance, changing patterns of hospitalization, non-uniform benefits across insurers and providers, and fragmented tariff arrangements.

**Policy options:**

Three policy directions emerge: modernizing VHI through innovation, strengthening communication with policyholders and patients, and improving public reporting on coverage, pricing, and use.

**Conclusion:**

Strengthening transparency while enabling innovation is essential if VHI is to remain a credible and sustainable component of the Swiss healthcare system.

## Background

Voluntary hospitalization insurance (VHI) in Switzerland provides additional coverage for inpatient care beyond the benefits guaranteed by mandatory health insurance (MHI). VHI is governed by private law–the Federal Insurance Contract Act (VVG/LCA) - and supervised by the Swiss Financial Market Supervisory Authority (FINMA) [1]. Notably, Swiss law does not allow VHI to substitute MHI or to cover copayments of the latter. Typical benefits include: 1) free choice of treating doctor during hospitalization; 2) additional comfort in a hospital room (private or semi-private ward); 3) larger choice of hospital [2]. In addition to these key benefits, some VHI products provide other benefits (like an alternative or innovative medical technique/product inpatient treatment abroad, accompanying person during the hospitalization, home nursing care after hospitalization, household help, childcare during the hospitalization, transportation to and from a hospital, and search/rescue/repatriation) [3].

The role of VHI has evolved considerably over time. Following the introduction of MHI in 1996, the scope of publicly regulated health coverage expanded [4, 5], reducing the relative importance of supplementary insurance. A further structural shift occurred with the 2012 hospital financing reform, which introduced cantonal hospital lists and required cantons to cover 55% of inpatient costs for listed providers [6, 7]. This reform effectively replaced part of the financing previously covered by VHI and contributed to its declining share in overall hospital financing. As shown in Figure 1 and in Figure 2, the contribution of voluntary insurance to hospital financing has steadily declined over time. The most pronounced drop occurred in 2012, immediately after the hospital financing reform. After 2012, the decline continued at a slower but steady pace in most hospital categories, while the VHI contribution increased slightly in psychiatric hospitals and more visibly in rehabilitation clinics (Figure 3).

**FIGURE 1:**
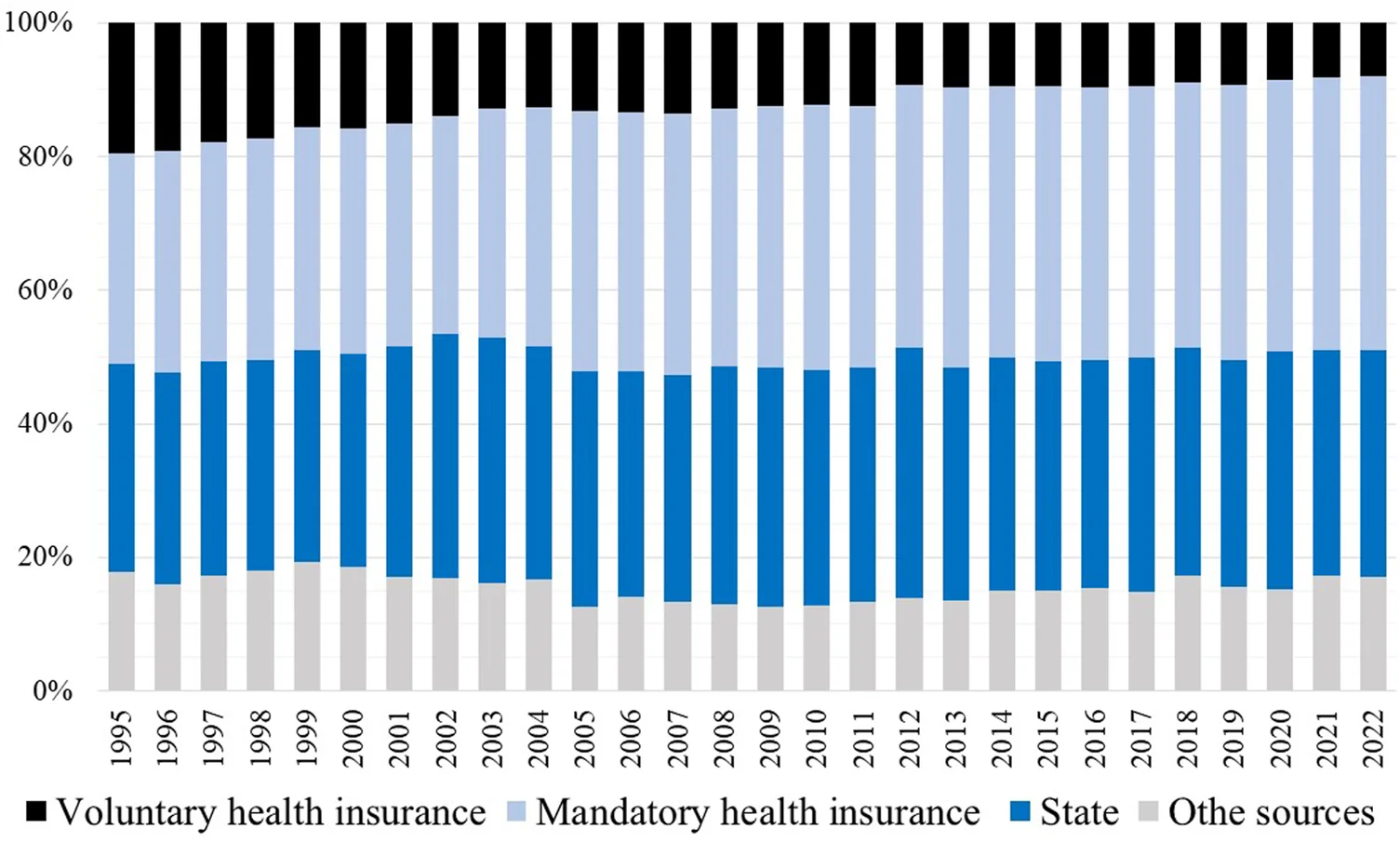
Hospitals financing by source (Switzerland, 1995–2022). Source: Authors’ calculations and visualization based on data from the Federal Statistical Office, “Cost of the healthcare system by provider and by financing scheme, 2024” [8]. Data were extracted from the original dataset, aggregated by category in Microsoft Excel, and then represented graphically.

**FIGURE 2:**
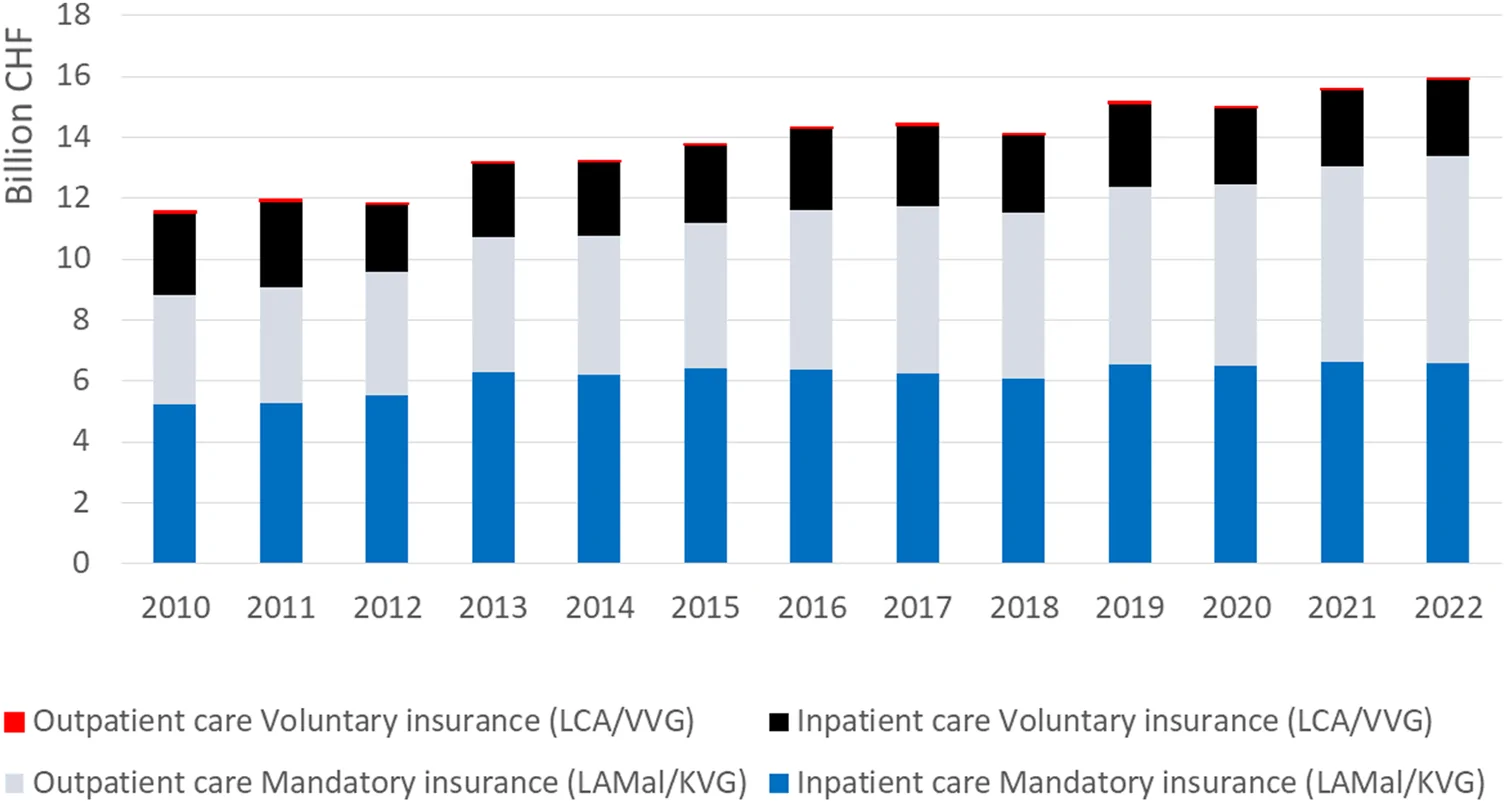
Trends in financing of inpatient and outpatient hospital services (Switzerland, 2010–2022). Source: Authors’ calculations and visualization based on data from the Federal Statistical Office, “Cost of the healthcare system by provider and by financing scheme, 2024” [8]. Data were extracted from the original dataset, aggregated by category in Microsoft Excel, and then represented graphically.

**FIGURE 3:**
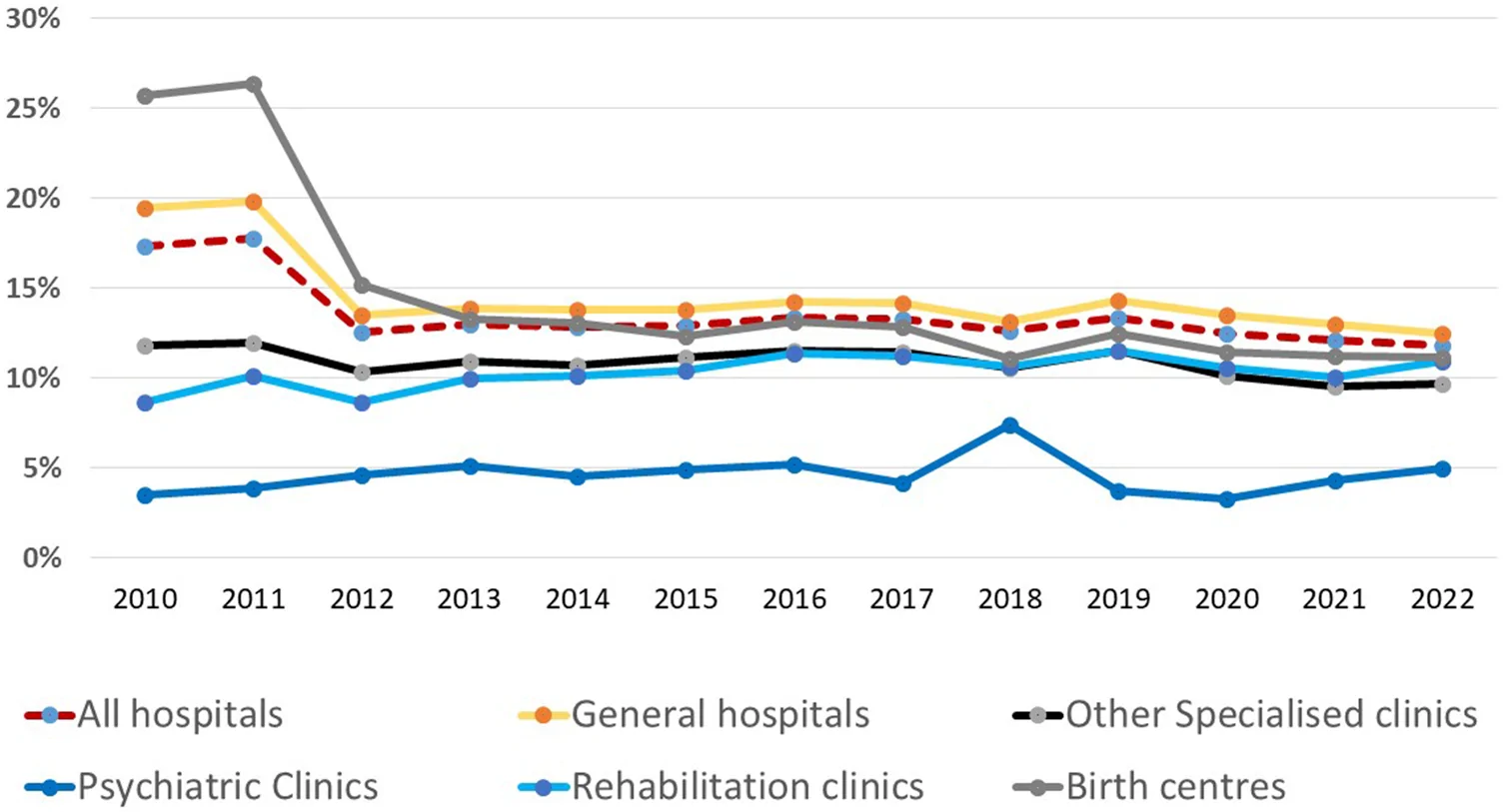
Share of inpatient hospital financing covered by voluntary hospitalization insurance, by hospital type, (Switzerland, 2010–2022). Source: Authors’ calculations and visualization based on data from the Federal Statistical Office, “Cost of the healthcare system by provider and by financing scheme, 2024” [8]. Data were extracted from the original dataset, aggregated by category in Microsoft Excel, and then represented graphically.

Despite this declining contribution, VHI remains a critical revenue stream within the Swiss healthcare system. For many decades, the Swiss health system has been under pressure of constantly raising health costs, and the hospital sector is not immune from this problem either [3, 9, 10]. In 2023, the Swiss Association of Public and Private Hospitals (H+) was alarmed about the insufficient hospital financing from MHI [11]. VHI remains strategically important in the current context of tight hospital financing [11–14]. As an additional source of financing, it diversifies financial flows and eases some of the pressure on hospitals.

Beyond providing the additional coverage for policyholders/insured and its direct contribution to hospital financing, VHI plays a central role in shaping financial incentives and revenue structures across key actors in the Swiss healthcare system. For insurers, VHI represents one of the few domains where profit generation is permitted [15]. For hospitals—and particularly for hospital head physicians—VHI revenues are closely associated with higher tariffs and supplementary fees linked to privately insured patients. These arrangements may create incentives to prioritize VHI patients, influence clinical decision-making and service provision, and affect the internal organization of care delivery [16].

However, these financial and incentive structures have increasingly been accompanied by concerns regarding transparency, pricing practices, and the additional value of VHI products. These concerns are highlighted in recent regulatory and policy reports. In December 2020, the Swiss Financial Market Supervisory Authority (FINMA) published a report indicating the need for comprehensive action regarding settlement practices, pointing to significant shortcomings in cost transparency and in the definition of “additional benefits” under VHI [17]. The report found that some medical bills issued by doctors and hospitals were unreasonably high or insufficiently justified, and in several cases it remained unclear what additional services were being charged beyond those already covered by mandatory health insurance. Similar concerns are raised by the Swiss federal price watchdog, whose analysis found that additional services for VHI patients generated average extra costs of CHF 6,745 per case in semi-private wards and CHF 8,960 in private wards, in addition to the costs covered by MHI. Prices varied substantially across providers, with the most expensive hospitals charging up to nine times more than the least expensive. The report also found that some pricing agreements automatically generated higher remuneration for the treatment of VHI patients even where no clinically differentiated services were provided [18].

## Analysis

This analysis combines evidence from a structured review of academic and grey literature, a desk review of regulatory and policy documents, a market assessment of insurance products, and qualitative stakeholder input. The regulatory review examined official documents published between 2009 and 2024 to assess the legal and institutional framework governing voluntary hospitalization insurance in Switzerland, while the market assessment drew on publicly available insurer information to analyse product features and market practices. These sources were complemented by secondary data from regulatory and mediation bodies (FINMA and Office de Médiation de l'Assurance-Maladie (OM-KV), as well as ten semi-structured interviews with key stakeholders, including policymakers, insurers, healthcare providers, and consumer representatives. Written communication with FINMA and the consumer organization Associazione consumatrici e consumatori della Svizzera italiana, based in the Italian‐speaking canton of Ticino in southern Switzerland, was also included in the analysis.

The future of VHI in Switzerland is shaped by four main factors: the expanding scope of MHI, changing patterns of hospitalization, the lack of standardization in VHI benefits, and the persistence of multiple tariff schemes.

### Dependence on the mandatory health insurance

Over the past 3 decades, the expansion of MHI has left less room for VHI. The 2012 hospital financing reform was a key turning point: by requiring cantons to establish hospital lists and cover 55% of inpatient costs for listed public and private hospitals, it replaced a significant share of financing that had previously been borne by VHI. In addition, since 2012, MHI has also covered hospital stays outside the canton of residence under limited conditions [7]. At the same time, rising MHI premiums have increased financial pressure on households, reducing their ability or willingness to purchase supplementary insurance.

### Changing approach to hospitalization

Broader changes in healthcare delivery are reducing the relevance of traditional hospitalization insurance. Inpatient care is increasingly being replaced by outpatient treatment, hospital stays are becoming shorter, and technological and demographic changes are transforming patterns of hospital use. As a result, there are objectively fewer opportunities to use conventional VHI products centered on inpatient hospitalization.

### Non-uniform benefits of VHI across insurers and health service providers

A further challenge for VHI is the lack of standardization in the benefits offered by insurers and hospitals, which makes informed consumer choice difficult. Insurers have considerable freedom in designing supplementary insurance products, including the services covered, the level of reimbursement, and any exclusions or limits [17]. Unlike mandatory health insurance, whose benefit package is clearly defined by regulation, VHI operates through individualized contracts that vary widely across insurers. While this flexibility may allow products to be tailored to different needs, it also makes policies difficult to compare and can weaken transparency for consumers.

Variation also exists on the provider side. Hospitals define their own catalogues of services for semi-private and private patients, and there is no official standard specifying what such categories should include. As a result, similar products may provide different benefits depending on the insurer and the hospital involved. In response to concerns raised by FINMA, the insurance industry introduced general principles in 2021 to clarify the concept of “additional benefits” under VHI [19]. However, these principles remain broad and do not establish a uniform standard, leaving important differences in benefits and service content unresolved.

### Multiple tariff systems for hospitalization insurance

Tariffs and reimbursement under VHI add further complexity to the system. Unlike MHI, where tariff structures are more tightly regulated, VHI operates largely under private law, allowing hospitals and physicians considerable freedom in defining supplementary tariffs. As a result, insurers negotiate separate pricing agreements with individual hospitals, hospital groups, and physicians, using different methods to calculate costs and bill services. This leads to limited transparency, substantial price variation, and considerable difficulty for policyholders in understanding invoices or assessing whether charges are justified [18]. Although insurers have simplified some tariff systems in recent years under pressure from FINMA, fragmented tariff arrangements remain a defining feature of the sector.

### Synthesis

Taken together, these four factors point to a central tension in the current VHI landscape. On the one hand, structural changes in health financing and care delivery have narrowed the space in which traditional hospitalization insurance can provide distinct value. On the other hand, the sector remains financially and strategically important for insurers, hospitals, and physicians. The future relevance of VHI will therefore depend on its ability to adapt to a changing healthcare environment, to offer more clearly defined and transparent benefits, and to reduce the complexity that currently characterizes product design and tariff arrangements.

### Limitations

This study has several limitations. It draws partly on secondary sources, which do not always permit independent verification of all claims discussed. The qualitative component involved a limited number of interviewees and stakeholder dialogue participants, and direct input from the Swiss Financial Market Supervisory Authority was not obtained. The recommendations are evidence-informed and stakeholder-informed, but they were ultimately synthesized by the researcher and should not be interpreted as a formally endorsed consensus.

## Policy options

The recommendations presented in this policy brief were developed through a combination of evidence review and stakeholder deliberation. They were informed by academic literature, regulatory and policy documents, market analysis, and qualitative research, and were further shaped by a stakeholder dialogue held at the University of Lucerne on 29 October 2024. The dialogue was organized by the Swiss Learning Health System and SUPSI and brought together national-level representatives of insurers, healthcare providers, and researchers. Moderated by two researchers, the discussion was structured around a SWOT analysis of voluntary hospitalization insurance in Switzerland and a focused exchange on barriers and facilitators for innovation and greater transparency in the sector. The recommendations presented below emerged from this multi-stakeholder dialogue and their practical implications are summarized in Table 1.

**TABLE 1:** Main actors and beneficiaries involved in implementing the proposed recommendations for voluntary hospitalization insurance in Switzerland, 2024.

​Recommendation	Main actors	Final beneficiaries
Recommendation 1: Modernizing VHI through innovation	- Insurers and health service providers for development and implementation of innovations - FINMA and policymakers on cantonal and federal levels for creating regulatory and institutional context that welcomes and enables innovations	- Policyholders and patients with new insurance products that have better fit to their needs - Insurers with new market opportunities and higher profit
Recommendation 2: Enhancing communication with policyholders and patients	- Insurers and health service providers for implementing better practices of communication with policyholders and patients - Insurers association for developing information materials for policyholders and patients that put together main elements (benefits, co-payment level, prices) from different insurers and hospitals/clinics in a clear, user-friendly, easy to read and compare way - Patient organizations, consumer organizations for dissemination of the info materials to the final beneficiaries	- Policyholders and patients with comprehensible, comparable information, that allows to make informed decisions and choices - Insurers and hospitals and clinics with higher satisfaction and trust from their clients
Recommendation 3: Improving public reporting on VHI	- Insurers and health service providers for providing more information on hospitalization insurance sector coverage and use - FINMA for creating and maintaining a proper data collection and monitoring system	- Policyholders and patients with clearer and more transparent VHI market - Insurers with higher trust from policyholders and regulators - FINMA, policymakers, and researchers with more information for analysis and market monitoring

### Recommendation 1. Modernizing voluntary hospitalization insurance through innovation

To remain relevant in a changing healthcare environment, VHI needs to move beyond largely traditional hospitalization products and support innovation in financing mechanisms, product design, and cooperation with healthcare providers. A first avenue for innovation concerns financing mechanisms. One option would be to introduce an individual savings component within VHI premiums, drawing on principles used in life insurance. Under such a model, part of the premium could accumulate over time and later be used to offset premium increases at older ages or facilitate switching between insurers. This approach could improve affordability across the life course and make coverage more accessible for older adults and individuals with pre-existing conditions. At the same time, such innovations would need to be assessed carefully within the existing legal and supervisory framework, including potential implications for product approval and regulatory oversight. A relevant real-world example of linking insurance with a savings mechanism is Singapore’s health financing model, where Medisave (mandatory medical savings accounts) can be used alongside Integrated Shield Plans, which provide supplementary hospital coverage beyond the basic public scheme [20].

A second avenue concerns product innovation. Future VHI products could move beyond room category and conventional inpatient privileges to include services better aligned with contemporary care pathways, such as post-hospitalization support, preventive services delivered in hospital settings, or selected forms of outpatient hospital care. More modular products could also improve flexibility and better match heterogeneous consumer preferences. A useful European example is the Dutch supplementary insurance market, where health insurers offer a wide range of modular or semi-modular supplementary products in addition to mandatory coverage [21].

A third area is innovation through insurer–provider collaboration. Stronger cooperation between insurers and healthcare providers could support preferred provider arrangements, integrated care models, or value-based approaches linking payment to quality and outcomes. However, such developments remain difficult in a fragmented system characterized by limited trust, regulatory pressure, and concerns about unjustified billing. Oversight remains essential to protect policyholders, but regulatory interventions should be designed in a way that preserves room for constructive cooperation and innovation. One of the clearest international examples of such collaboration is Kaiser Permanente in the United States, which is often cited as a model of integrated financing and service delivery. Insurance, provider networks, and care coordination are brought together in a more unified structure than in fragmented systems [22].

### Recommendation 2. Enhancing communication with policyholders and patients

Improving communication with policyholders and patients should be a priority across all stages of the insurance pathway [23, 24]. At the point of enrollment, information on coverage, exclusions, and expected costs should be presented in a clear, accessible, and comparable way. During hospitalization planning, patients should receive transparent information on which services are covered by VHI, which remain within the scope of mandatory health insurance, and whether additional charges may apply. After hospitalization, invoices should be sufficiently detailed and understandable to allow policyholders to identify what has been billed and on what basis.

Better communication would improve informed decision-making, reduce uncertainty, and strengthen confidence in the sector. It would also help address one of the central weaknesses identified in the analysis: the difficulty for policyholders to understand the actual content and value of VHI coverage.

### Recommendation 3. Improving public reporting on hospitalization insurance

Greater transparency in the VHI sector requires stronger public reporting. Systematic publication of comparable information on coverage, pricing, and use of VHI would improve accountability, support public oversight, and enable more informed consumer choice [25–27]. Public reporting could also help identify variation in practices across insurers and providers and provide a stronger basis for policy debate.

A practical first step would be the development of an official statistical reporting framework for VHI. Such a framework could include the number of people covered by hospitalization insurance, disaggregated by age, gender, and canton of residence; the type and price of coverage; and selected indicators on the use of VHI benefits. In a second phase, reporting could be expanded to include information on reimbursement patterns, billing practices, or provider-level variation. Depending on the scope of the system, responsibility for data collection and publication could lie with institutions such as the Federal Office of Public Health, FINMA, or the Swiss Health Observatory.

## Conclusion

Voluntary hospitalization insurance in Switzerland is at a critical juncture. Its traditional role has declined as mandatory health insurance has expanded and patterns of hospitalization have changed, yet it remains financially and strategically important for several actors in the health system. At the same time, concerns about transparency, pricing, and the comparability of benefits continue to weaken its value for policyholders.

To remain relevant, VHI must adapt to this changing environment. This requires both modernization of products and financing mechanisms, and stronger transparency through clearer communication and improved public reporting. A balanced regulatory approach will be essential to protect consumers while allowing the innovation needed for VHI to remain a credible and sustainable component of the Swiss healthcare system.
